# Supplementary material for: Cytological and molecular characterizations of a novel 2A nullisomic line derived from a widely-grown wheat cultivar Zhoumai 18 conferring male sterility
Source: PeerJ. 2020 Oct 30;8:e10275. doi: 10.7717/peerj.10275 (PMC7605228; doi:10.7717/peerj.10275)
Supplement: Supplemental Information S1 [file peerj-08-10275-s001.doc]

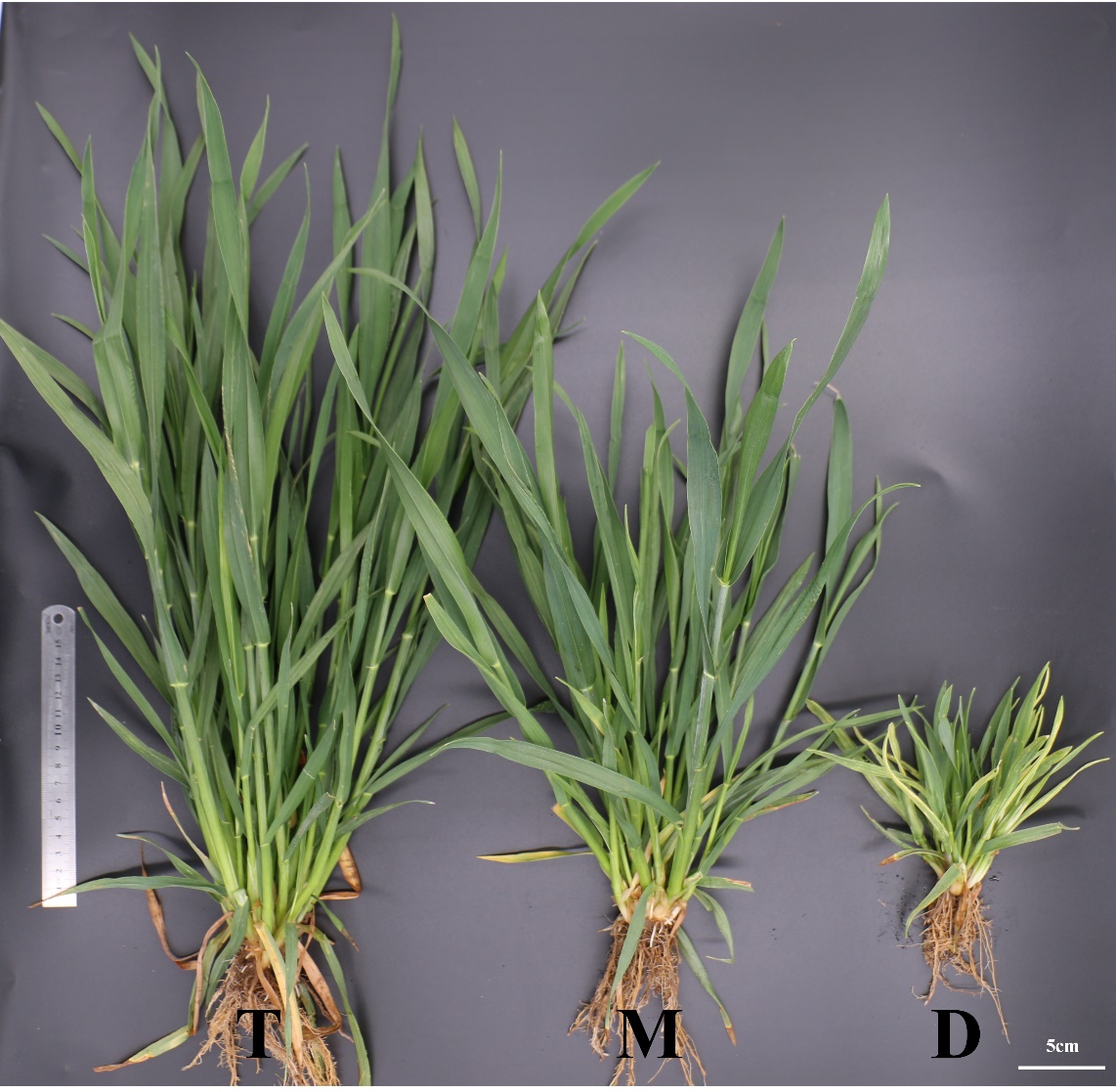


**Figure S1** The tall (T), semi-dwarf (M) and dwarf (D) individuals of *dms*.


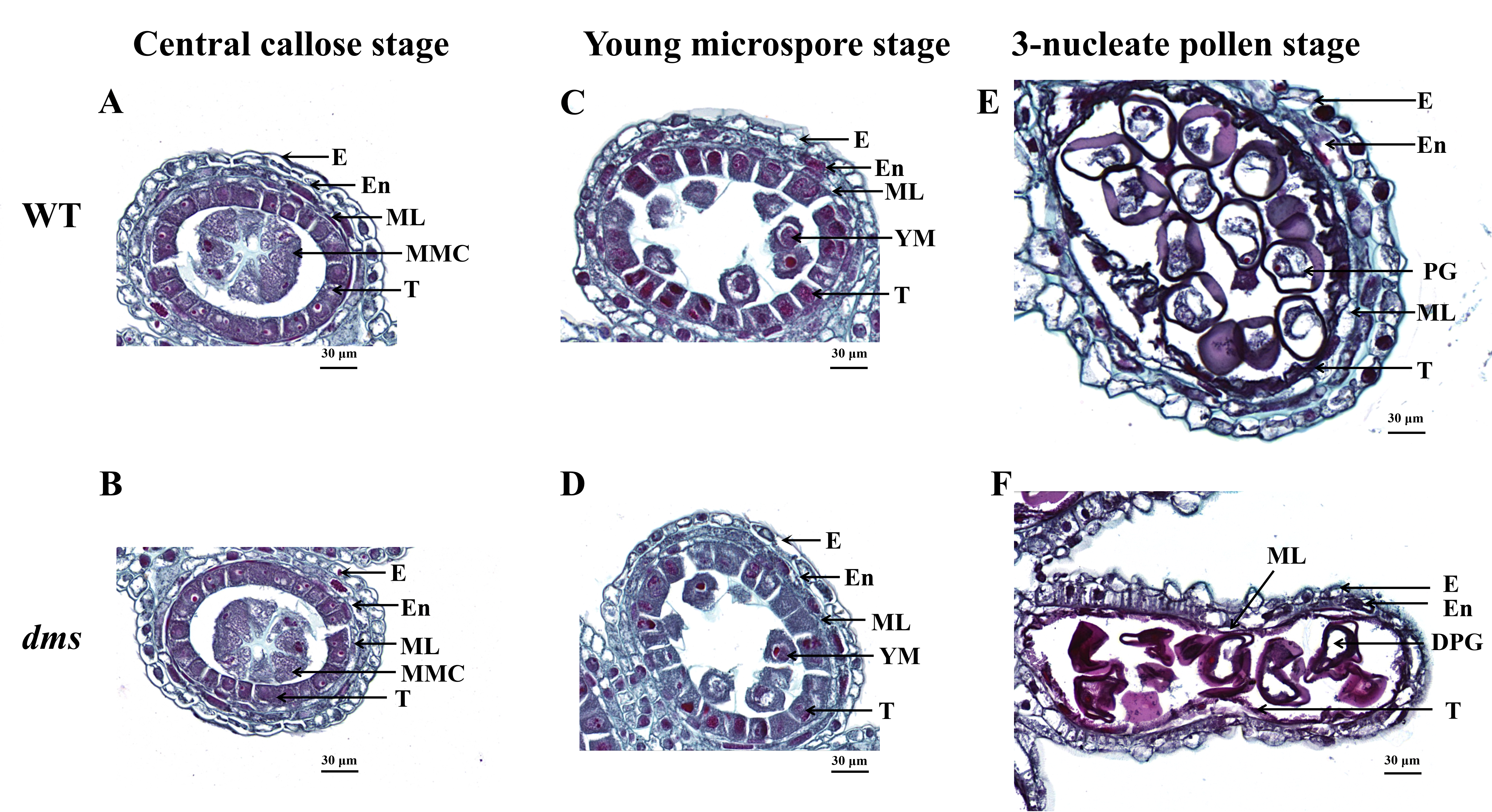


**Figure S2** Transverse section analysis of the anther development in wild-type (A, C and E) and *dms* mutant (B, D and F). (A) and (B) Central callose stage; (C) and (D) Young microspore stage; (E) and (F) 3-nucleate pollen stage. E, Epidermis; En, Endothecium; ML, Middle Layer; T, Tapetum; MMC, Microspore Mother Cells; YM, Young Microspores; PG, Pollen Grains; DPG, Degenerated Pollen Grains. Scale bars: 30 μm.


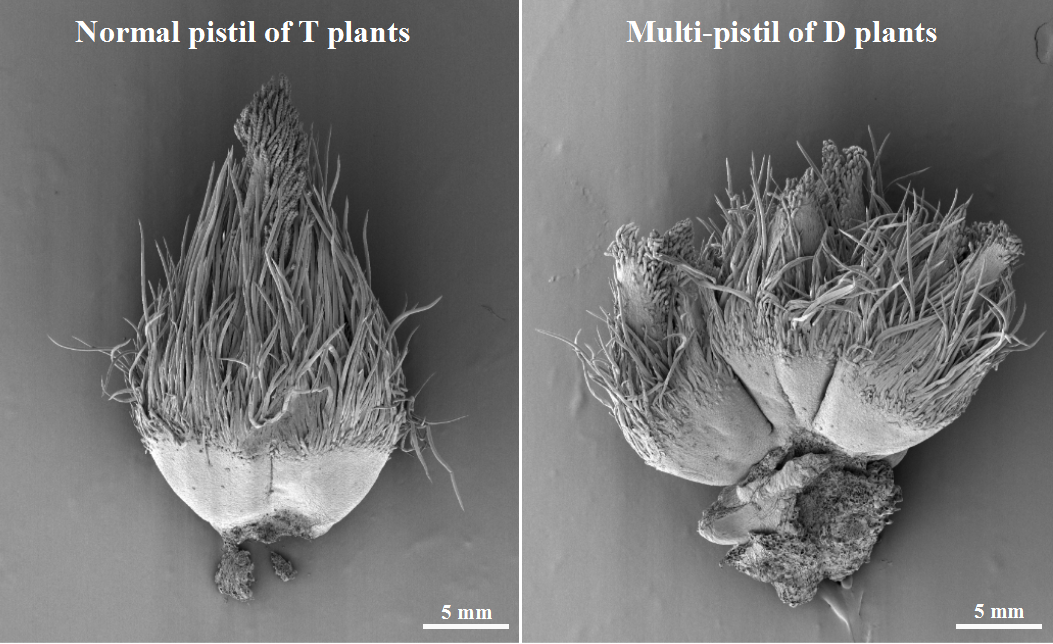


**Figure S3** The scanning electron micrographs of the pistils in T (left) and D (right) plants of *dms*.


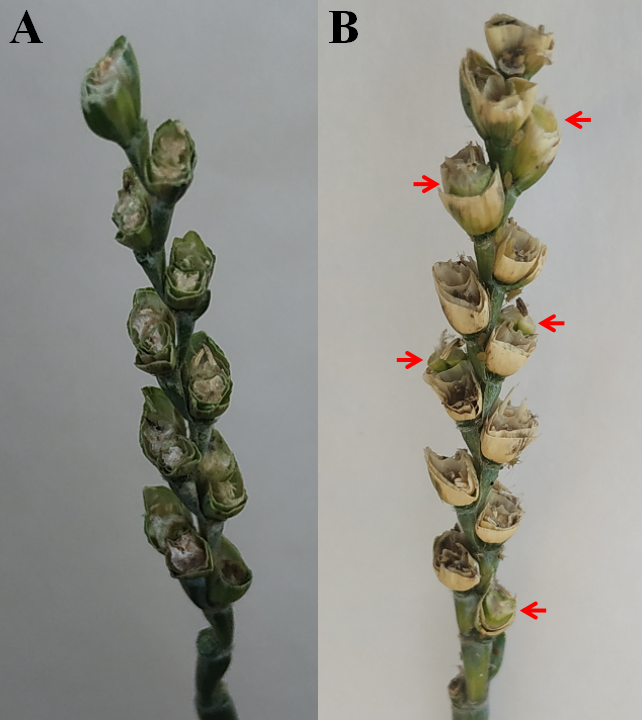


**Figure S4** The D plants were male sterile but female fertile. (**A**) The D plants were male sterile. (**B**) The set seeds in the spikelets of D (as female parent) plants pollinated with pollens of Guomai 301 (as male parent). The seeds had set for 2 weeks after pollination.


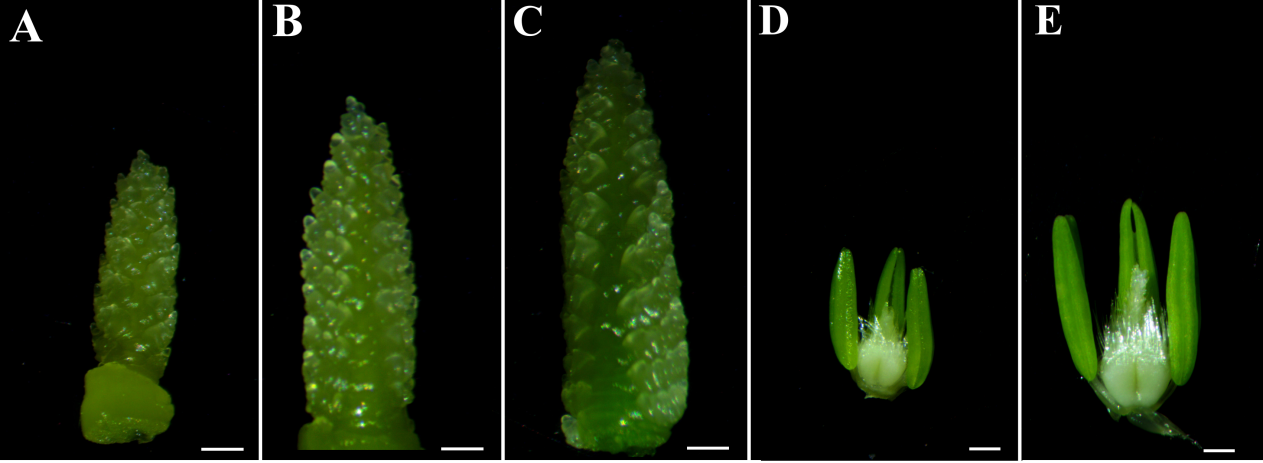


**Figure S5** The samples used for RNA-Sequencing. (**A-C**) The young spikes of D, M and T plants at floret primordium visible stage, (**D** and **E**) The anthers of D and T plants at 3-nucleate pollen stage.


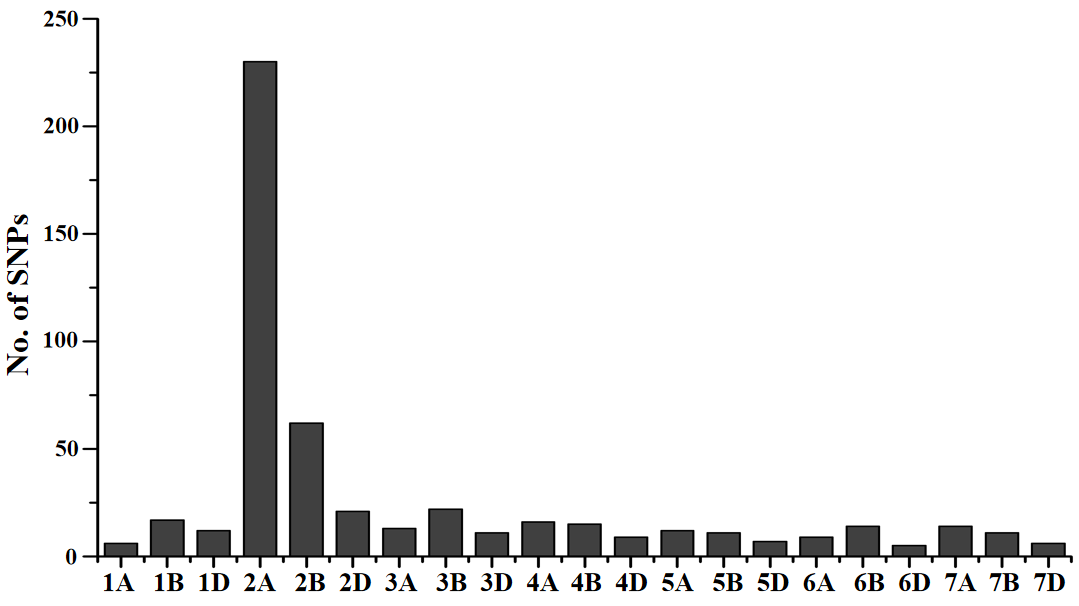


**Figure S6** Chromosome distribution of the SNPs between the stamens of T and D plants.


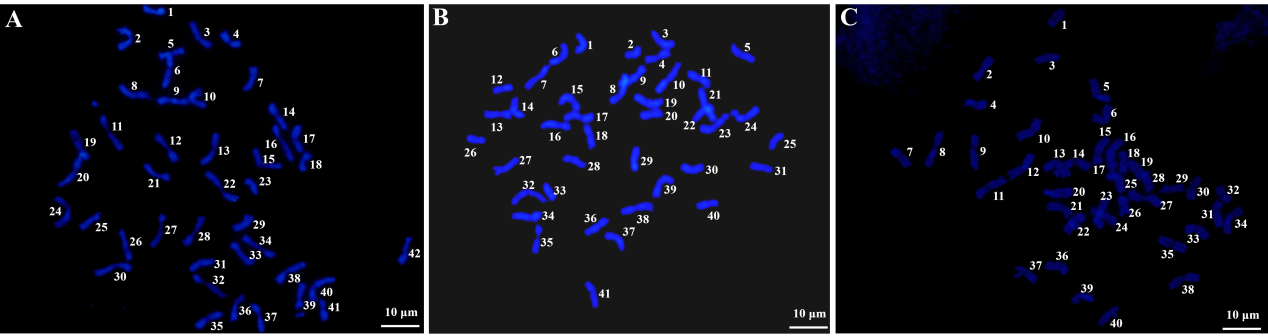


**Figure S7** Chromosome configurations of the plants derived from M plants at mitosis metaphase. **(A)** A karyotype with 42 normal chromosomes. **(B)** A karyotype with 41 chromosomes, lacked one chromosome. **(C)** A karyotype with 40 chromosomes, lacked two chromosomes.


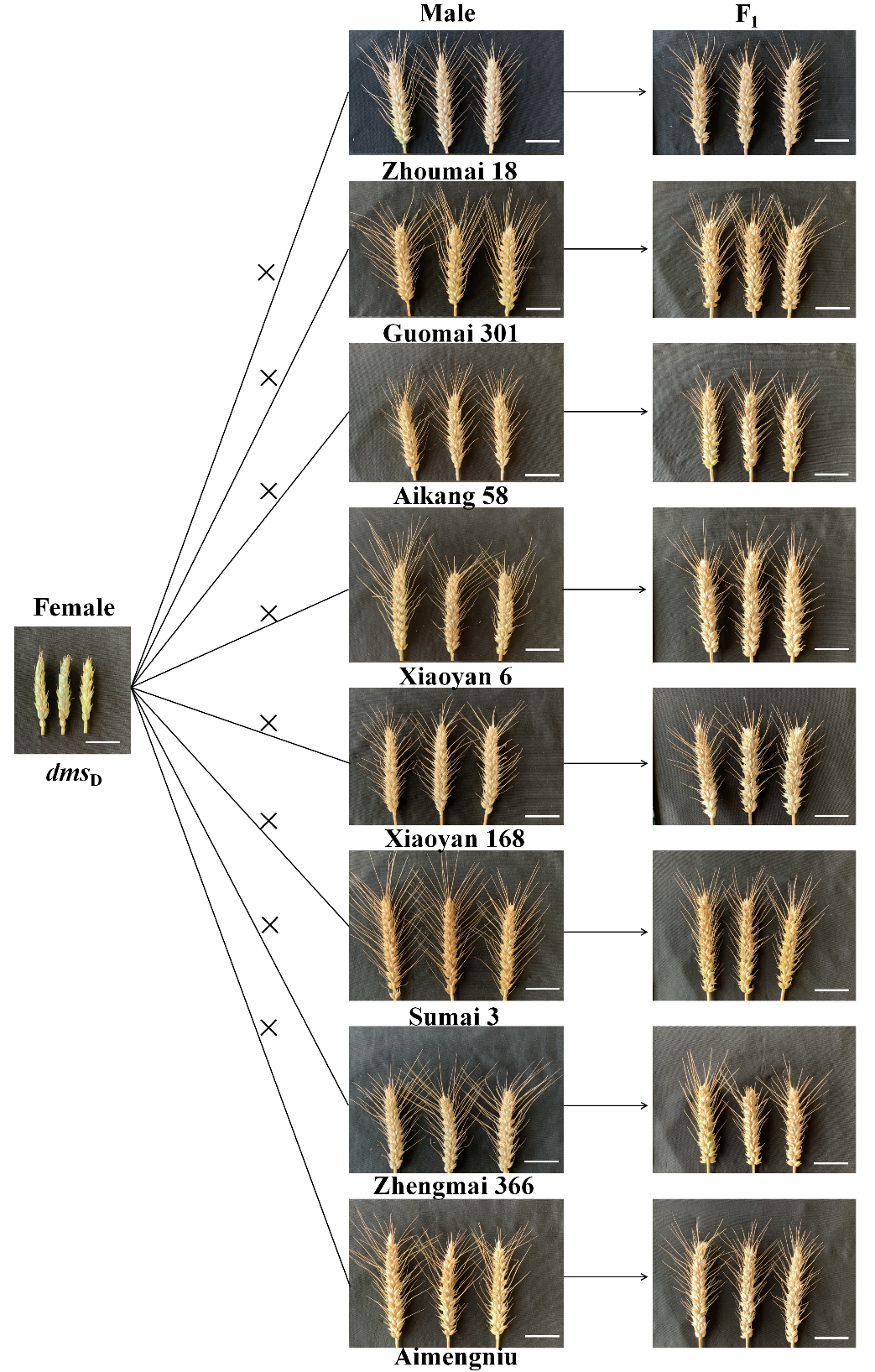


**Figure S8** Spike phenotypes of the 8 crosses at F_1_ generations with D plant as female and the other wheat genotypes as pollen parent.

**
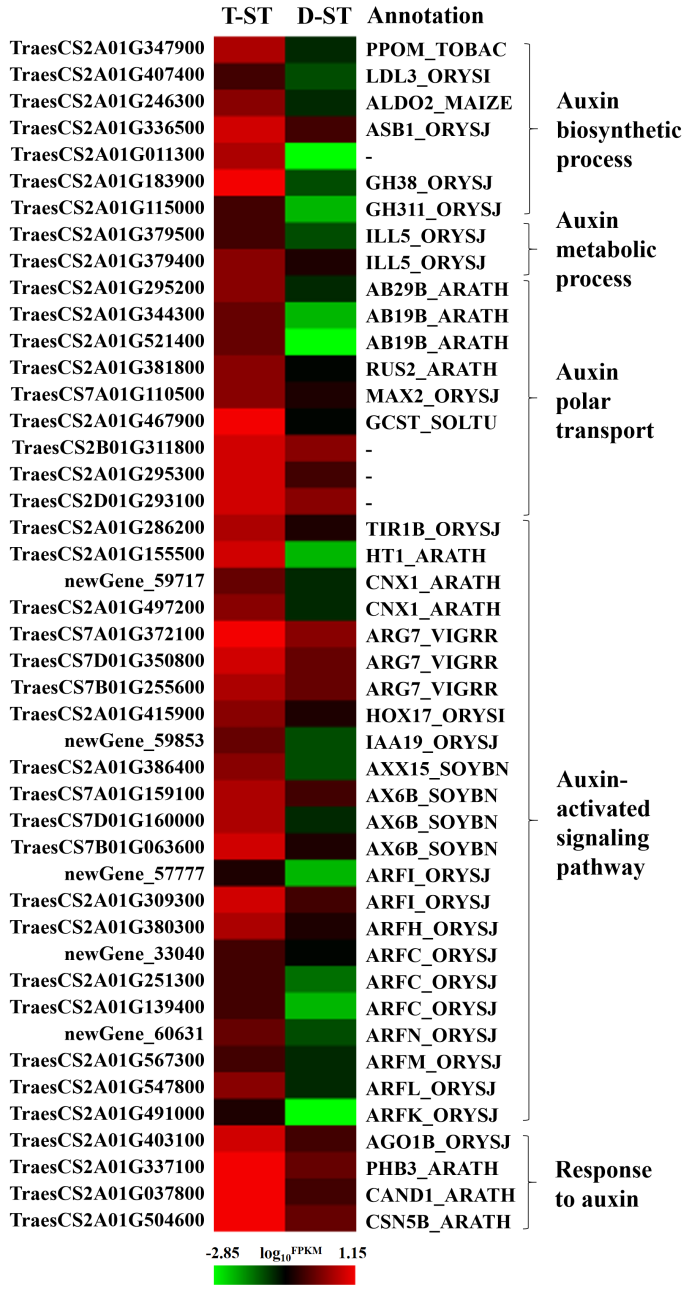
**

**Figure S9** Heatmap of auxin homeostasis related DEGs.
